# Six-year outcomes of robot-assisted radical prostatectomy versus volumetric modulated arc therapy for localized prostate cancer: A propensity score-matched analysis

**DOI:** 10.1007/s00066-023-02192-5

**Published:** 2024-01-05

**Authors:** Michio Noda, Satoru Taguchi, Kenshiro Shiraishi, Tetsuya Fujimura, Akihiro Naito, Taketo Kawai, Jun Kamei, Yoshiyuki Akiyama, Yuta Yamada, Yusuke Sato, Daisuke Yamada, Tohru Nakagawa, Hideomi Yamashita, Keiichi Nakagawa, Osamu Abe, Hiroshi Fukuhara, Haruki Kume

**Affiliations:** 1 Department of Urology, Graduate School of Medicine, The University of Tokyo, 7-3-1 Hongo, Bunkyo-ku, 113-8655 Tokyo, Japan; 2 Department of Urology, Teikyo University School of Medicine, Tokyo, Japan; 3 Department of Radiology, Teikyo University School of Medicine, Tokyo, Japan; 4 Department of Radiology, The University of Tokyo Hospital, Tokyo, Japan; 5 Department of Urology, Jichi Medical University, Tochigi, Japan; 6 Department of Urology, Kyorin University School of Medicine, Tokyo, Japan

**Keywords:** External beam radiotherapy, Intensity-modulated radiotherapy, Prostate cancer, Robot-assisted radical prostatectomy, Volumetric modulated arc therapy

## Abstract

**Background:**

Although robot-assisted radical prostatectomy (RARP) and intensity-modulated radiotherapy are the leading respective techniques of prostatectomy and radiotherapy for localized prostate cancer, almost no study has directly compared their outcomes; none have compared mortality outcomes.

**Methods:**

We compared 6‑year outcomes of RARP (*n* = 500) and volumetric modulated arc therapy (VMAT, a rotational intensity-modulated radiotherapy, *n* = 360) in patients with cT1-4N0M0 prostate cancer. We assessed oncological outcomes, namely overall survival (OS), cancer-specific survival (CSS), radiological recurrence-free survival (rRFS), and biochemical recurrence-free survival (bRFS), using propensity score matching (PSM). We also assessed treatment-related complication outcomes of prostatectomy and radiotherapy.

**Results:**

The median follow-up duration was 79 months (> 6 years). PSM generated a matched cohort of 260 patients (130 per treatment group). In the matched cohort, RARP and VMAT showed equivalent results for OS, CSS, and rRFS: both achieved excellent 6‑year outcomes for OS (> 96%), CSS (> 98%), and rRFS (> 91%). VMAT had significantly longer bRFS than RARP, albeit based on different definitions of biochemical recurrence. Regarding complication outcomes, patients who underwent RARP had minimal (2.6%) severe perioperative complications and achieved excellent continence recovery (91.6 and 68.8% of the patients achieved ≤ 1 pad/day and pad-free, respectively). Patients who underwent VMAT had an acceptable rate (20.0%) of grade ≥ 2 genitourinary complications and a very low rate (4.4%) of grade ≥ 2 gastrointestinal complications.

**Conclusion:**

On the basis of PSM after a 6-year follow-up, RARP and VMAT showed equivalent and excellent oncological outcomes, as well as acceptable complication profiles.

**Supplementary Information:**

The online version of this article (10.1007/s00066-023-02192-5) contains supplementary material, which is available to authorized users.

## Introduction

Radical prostatectomy and external beam radiotherapy are comparable treatment options for localized prostate cancer (PC) [1–3]. Although many studies have compared oncological outcomes, the issue of which treatment is better remains controversial [3–18]. Notably, outcomes of the Prostate Testing for Cancer and Treatment (ProtecT) trial have recently been updated, with comparable mortality outcomes following prostatectomy and radiotherapy as well as active monitoring at a median follow-up of 15 years [18].

Robot-assisted radical prostatectomy (RARP) and intensity-modulated radiotherapy (IMRT) are the leading respective techniques for prostatectomy and radiotherapy, both of which are currently used worldwide. Nevertheless, almost no study has compared outcomes between these methods. We previously reported the comparative outcomes of RARP versus volumetric modulated arc therapy (VMAT), a sophisticated radiotherapy technique based on rotational IMRT [19], in 860 patients with cT1-4N0M0 PC [12]. Although it was the first (and currently the only) study that compared the outcomes of RARP and IMRT, the follow-up duration (median: 37 months) was too short to compare mortality outcomes. Therefore, the present study extended the follow-up duration by 5 years from that in the previous study [12]. In the present study, we compared the patients’ outcomes, including mortality rates, using propensity score matching (PSM) after a follow-up duration of > 6 years. We also assessed the complication outcomes of RARP and VMAT after the extended follow-up.

## Methods and materials

### Patients and treatments

This retrospective study was approved by the internal institutional review board of the Graduate School of Medicine and Faculty of Medicine, The University of Tokyo (approval number: 12003). All methods were conducted in accordance with the 1964 Declaration of Helsinki and its later amendments or comparable ethical standards. Given the retrospective nature of this study, the requirement for written informed consent was waived. We also obtained permission to reuse the previously published material [12] from Elsevier (License number: 5571801413900).

The patient selection process was described in our previous article [12]. Briefly, we reviewed data for 874 patients with PC who underwent either RARP (*n* = 500) or VMAT (*n* = 374) with curative intent at The University of Tokyo Hospital between 2011 and 2016. After excluding 14 patients from the VMAT group (cN1M0 disease, *n* = 7; VMAT combined with brachytherapy, *n* = 7), we analyzed data for 860 patients with cT1-4N0M0 PC who underwent RARP (*n* = 500) or VMAT (*n* = 360) [12]. Patients were stratified in accordance with the D’Amico risk classification [4], and the age-adjusted Charlson comorbidity index (CCI) was evaluated at baseline [20].

Details of the treatments are described in our previous article [12]. Briefly, RARP was performed using the peritoneal approach by modifying the Vattikuti Institute prostatectomy technique [21, 22]. Lymph node dissections were performed in those predicted to have ≥ 5% lymph node metastasis in accordance with the Japan PC nomogram [23]. Regarding VMAT, each treatment comprised a single-arc (from −179° to +179°, clockwise) using 6 MV X‑rays. The prescribed dose was 76 Gy in 38 fractions to cover 95% of the planning target volume, whereas the dose was limited to 72 Gy in 36 fractions in those receiving antithrombotic agents, to prevent rectal bleeding. Neoadjuvant androgen deprivation therapy (ADT) was administered for 4–6 months in intermediate-risk patients, and for 6 months in high- and very-high-risk patients. Adjuvant ADT was also administered to appropriate patients for 2–3 years, at the physician’s discretion.

### Endpoints and follow-up

Overall survival (OS), cancer-specific survival (CSS), radiological recurrence-free survival (rRFS), and biochemical recurrence-free survival (bRFS) were compared between the RARP and VMAT groups. OS was defined as the time from treatment initiation (the day of surgery for the RARP group and the starting date of radiotherapy for the VMAT group) to all-cause death. CSS was defined as the time from treatment initiation to cancer-specific death. Radiological recurrence was defined as radiologically-diagnosed distant metastasis or local recurrence. Biochemical recurrence was defined as two consecutive prostate-specific antigen (PSA) measurements ≥ 0.2 ng/ml for RARP [2] and the Phoenix definition (PSA ≥ nadir + 2 ng/ml) for VMAT [24], in accordance with the standard definition of biochemical recurrence for each modality. Recurrence-free survival (rRFS or bRFS) was defined as the time from treatment initiation to each defined recurrence or all-cause death, whichever occurred first. All patients were followed-up with routine blood tests, including PSA, every 1–6 months. With signs of biochemical recurrence, metastatic work-up by imaging studies, including computed tomography and bone scintigraphy, was routinely performed. Given that our previous study analyzed data obtained as of March 2018 [12], follow-up information for this study was obtained as of March 2023 (*i.e.,* the follow-up duration was extended for 5 years).

Aside from oncological outcomes, we collected data on treatment-related complications. For the RARP group, postoperative complications were categorized in accordance with the Clavien–Dindo classification [25, 26]. To evaluate continence recovery after surgery, patients were asked to remember the date when they achieved ≤ 1 pad per day or pad-free, data for which were prospectively collected in our database [27]. For the VMAT group, genitourinary (GU) and gastrointestinal (GI) adverse events were assessed using the Common Terminology Criteria for Adverse Events version 5.0.

### Statistical analysis

For PSM, multivariate logistic regression analysis was used to calculate propensity scores, and matching was performed on the logit of the propensity score using the nearest neighbor matching with a caliper width of 0.20. Before and after PSM, the significance of the differences in clinicopathological variables between the treatment groups were evaluated using Student’s *t*-test for continuous variables and the χ^2^ test for categorical variables. Before and after PSM, OS, CSS, rRFS, and bRFS were estimated using the Kaplan–Meier method and compared using the log-rank test. The Cox proportional hazard regression model was used for univariate and multivariate analyses for OS, rRFS, and bRFS before and after PSM (note: analyses for CSS could not be performed owing to the small number of events). Cumulative proportions of continence recovery in the RARP group and those of grade ≥ 2 GU and GI complications in the VMAT group were also estimated using the Kaplan–Meier method. All statistical analyses were performed using JMP Pro version 16.0.0 (SAS Institute, Cary, NC, USA). *P* < 0.05 was considered significant.

## Results

### Analyses of crude data before PSM

The left half of Table 1 shows the baseline characteristics of all patients (*n* = 860) before PSM. There were significant differences between the RARP and VMAT groups for all variables that were assessed. The median follow-up duration was 79 months (interquartile range [IQR]: 62–98 months) and those in the RARP and VMAT groups were 76 months (IQR: 63–89 months) and 87 months (IQR: 59–108 months), respectively (Student’s *t*-test, *P* < 0.001). All VMAT patients who underwent ADT received neoadjuvant ADT, which was continued if indicated: 88 of 360 (24.4%) VMAT patients underwent long-term (≥ 2 years) ADT. Detailed descriptions of neoadjuvant and/or adjuvant treatments in the RARP group are provided in our previous article [12].

**Table 1:** Patients’ baseline characteristics before and after PSM

Parameter	Before PSM			After PSM		
	RARP (*n* = 500)	VMAT (*n* = 360)	*P*	RARP (*n* = 130)	VMAT (*n* = 130)	*P*
*Age, years, median (IQR)*	67 (63–71)	71 (66–75)	< 0.001^*a^	70 (66–73)	69 (65–74)	0.49^a^
*Initial PSA, ng/ml, median (IQR)*	7.6 (5.6–11.1)	8.3 (5.8–16.7)	< 0.001^*a^	7.9 (5.8–11.8)	6.9 (5.4–9.6)	0.66^a^
*Biopsy Gleason score, no.* *(%):*	–	–	0.015^*b^	–	–	0.89^b^
6	94 (18.8)	58 (16.1)	–	27 (20.8)	26 (20.0)	–
7	284 (56.8)	189 (52.5)	–	83 (63.9)	83 (63.9)	–
8	73 (14.6)	51 (14.2)	–	8 (6.2)	6 (4.6)	–
≥ 9	49 (9.8)	62 (17.2)	–	12 (9.2)	15 (11.5)	–
*Clinical T stage, no.* *(%):*	–	–	< 0.001^*b^	–	–	0.99^b^
1c	393 (78.6)	175 (48.6)	–	89 (68.5)	88 (67.7)	–
2a	56 (11.2)	28 (7.8)	–	15 (11.5)	15 (11.5)	–
2b	23 (4.6)	43 (11.9)	–	12 (9.2)	13 (10.0)	–
2c	21 (4.2)	64 (17.8)	–	11 (8.5)	10 (7.7)	–
≥ 3	7 (1.4)	50 (13.9)	–	3 (2.3)	4 (3.1)	–
*D’Amico risk classification, no.* *(%):*	–	–	< 0.001^*b^	–	–	0.94^b^
Low	70 (14.0)	34 (9.4)	–	22 (16.9)	20 (15.4)	–
Intermediate	281 (56.2)	154 (42.8)	–	79 (60.8)	81 (62.3)	–
High	149 (29.8)	172 (47.8)	–	29 (22.3)	29 (22.3)	–
*CCI, median (IQR):*						
Comorbidity score	0 (0–0)	0 (0–2)	< 0.001^*a^	0 (0–0)	0 (0–0)	0.57^a^
Age score	3 (3–4)	4 (3–4)	< 0.001^*a^	3 (3–4)	3 (3–4)	0.22^a^
Total score (= age-adjusted CCI)	3 (3–4)	4 (3–5)	< 0.001^*a^	4 (3–4)	4 (3–4)	0.24^a^
*Concomitant ADT, no. (%)*	25 (5.0)	224 (62.2)	< 0.001^*b^	22 (16.9)	26 (20.0)	0.52^b^

*ADT* androgen-deprivation therapy, *CCI* Charlson comorbidity index, *IQR* interquartile range, *PSA* prostate-specific antigen, *PSM* propensity score matching, *RARP* robot-assisted radical prostatectomy, *VMAT* volumetric modulated arc therapy

^*^Statistically significant

^a^Student’s *t*-test

^b^χ^2^ test

Supplementary Fig. 1 shows the Kaplan–Meier curves of the RARP vs. VMAT patients for OS, CSS, rRFS, and bRFS in the original cohort before PSM (*n* = 860). In the RARP group, 111 patients developed biochemical recurrence, 18 developed radiological recurrence, 2 died of PC, and 11 died from other causes. In the VMAT group, 37 patients developed biochemical recurrence, 15 developed radiological recurrence, 3 died of PC, and 21 died from other causes. Accordingly, RARP patients had significantly longer OS compared with VMAT patients (log-rank test, *P* = 0.027; Supplementary Fig. 1A), whereas there were no significant differences between the groups for CSS (*P* = 0.54; Supplementary Fig. 1B) and rRFS (*P* = 0.14; Supplementary Fig. 1C). VMAT patients had significantly longer bRFS compared with RARP patients, albeit based on different definitions of biochemical recurrence (*P* < 0.001; Supplementary Fig. 1D). In addition, there was no difference in bRFS between VMAT with < 2-year ADT vs. VMAT with ≥ 2-year ADT (Supplementary Fig. 2A). For reference, 6‑year OS, CSS, rRFS, and bRFS rates in the RARP group were 97.6%, 99.6%, 95.3%, and 76.8%, while those in the VMAT group were 94.9%, 99.3%, 92.3%, and 89.5%, respectively.

Univariate and multivariate Cox proportional hazard regression analyses in all patients (*n* = 860) before PSM were performed for OS, rRFS, and bRFS, but not for CSS owing to the small number of events (*n* = 5) (Supplementary Table 1). In the multivariate analyses, D’Amico risk classification (low vs. intermediate vs. high) and age-adjusted CCI (continuous) were identified as independent prognostic factors for both OS (Supplementary Table 1A) and rRFS (Supplementary Table 1B), whereas treatment modality (RARP vs. VMAT) and concomitant ADT (yes vs. no) were not. In contrast, treatment modality was identified as an independent prognostic factor for bRFS, along with D’Amico risk classification and concomitant ADT (Supplementary Table 1C).

### Analyses after PSM

Table 1 shows the baseline characteristics of patients before and after PSM. For PSM, all seven variables listed in Table 1 were matched: age, initial PSA, biopsy Gleason score, clinical T stage, D’Amico risk classification, age-adjusted CCI, and concomitant ADT. The right half of Table 1 shows the characteristics of 260 patients after PSM. Baseline characteristics between the two groups (RARP vs. VMAT) were balanced after PSM, and differences between all variables were nonsignificant. Eight of 130 (6.2%) VMAT patients underwent long-term (≥ 2 years) ADT.

**Fig. 1:**
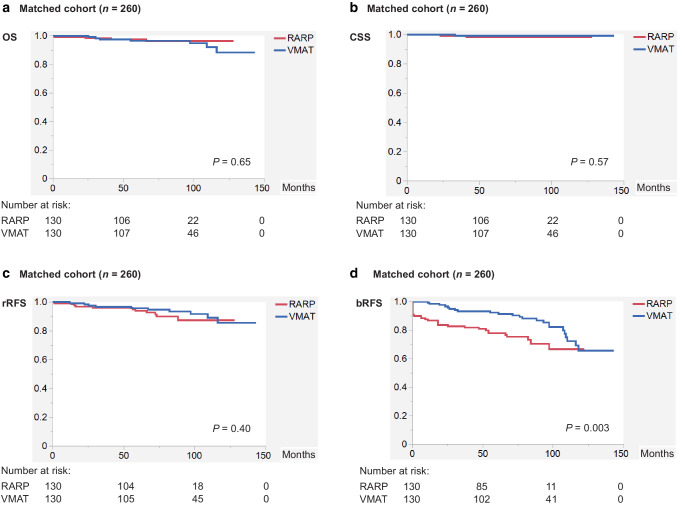
Kaplan–Meier curves of the RARP vs. VMAT patients for **a** OS, **b** CSS, **c** rRFS, and **d** bRFS in the matched cohort (*n* = 260). (*bRFS* biochemical recurrence-free survival, *CSS* cancer-specific survival, *OS* overall survival, *RARP* robot-assisted radical prostatectomy, *rRFS* radiological recurrence-free survival, *VMAT* volumetric modulated arc therapy)

Figure 1 shows the Kaplan–Meier curves of the RARP vs. VMAT patients for OS, CSS, rRFS, and bRFS in the matched cohort (*n* = 260). After PSM, 30 patients developed biochemical recurrence, nine developed radiological recurrence, two died of PC, and two died from other causes in the RARP group, whereas 17 patients developed biochemical recurrence, four developed radiological recurrence, one died of PC, and six died from other causes in the VMAT group. Accordingly, RARP and VMAT patients had equivalent outcomes for OS (log-rank test, *P* = 0.65; Fig. 1a), CSS (*P* = 0.57; Fig. 1b), and rRFS (*P* = 0.40; Fig. 1c), whereas VMAT patients had significantly longer bRFS compared with RARP patients based on different definitions of biochemical recurrence (log-rank test, *P* = 0.003; Fig. 1d). In addition, there was no difference in bRFS between VMAT with < 2-year ADT vs. VMAT with ≥ 2-year ADT (Supplementary Fig. 2B). For reference, 6‑year OS, CSS, rRFS, and bRFS rates in the RARP group were 96.4%, 98.3%, 91.6%, and 75.4%, while those in the VMAT group were 96.5%, 99.1%, 94.7%, and 90.4%, respectively.

Univariate and multivariate Cox proportional hazard regression analyses after PSM (*n* = 260) were performed for OS, rRFS, and bRFS, but not for CSS owing to the small number of events (*n* = 3) (Supplementary Table 2). No variable, including treatment modality (RARP vs. VMAT), was an independent prognostic factor on multivariate analysis for both OS (Supplementary Table 2A) and rRFS (Supplementary Table 2B), whereas treatment modality and concomitant ADT were independent prognostic factors for bRFS (Supplementary Table 2C).

### Complication outcomes of RARP and VMAT

**Fig. 2:**
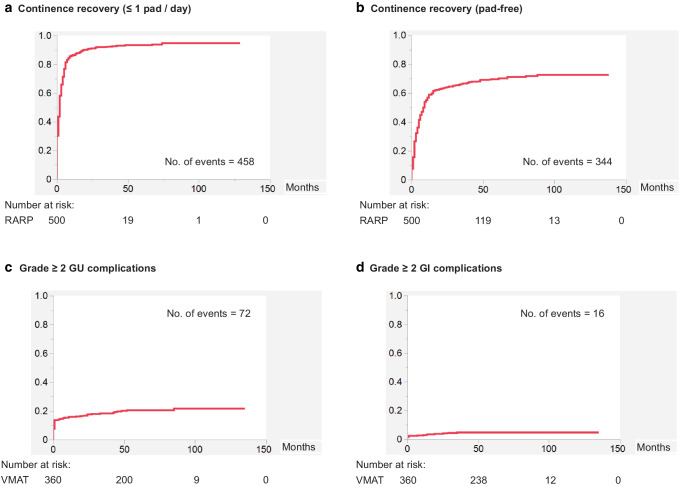
Cumulative proportions of continence recovery in the RARP group (*n* = 500) and those of grade ≥ 2 GU and GI complications in the VMAT group (*n* = 360). **a** Proportion of patients achieving ≤ 1 pad/day for urinary continence and **b** that of patients achieving pad-free status for urinary continence in the RARP group (*n* = 500). **c** Proportion of patients with grade ≥ 2 GU complications and **d** that of patients with grade ≥ 2 GI complications in the VMAT group (*n* = 360). (*GI* gastrointestinal, *GU* genitourinary, *RARP* robot-assisted radical prostatectomy, *VMAT* volumetric modulated arc therapy)

In addition to oncological outcomes, we assessed complication outcomes of RARP and VMAT. Supplementary Table 3 shows the Clavien–Dindo grade ≥ 3 perioperative complications in the RARP group (*n* = 500). Thirteen of 500 (2.6%) patients experienced grade ≥ 3 complications, such as postoperative hemorrhage (*n* = 3), rectal injury (*n* = 2), small bowel injury (*n* = 2), and abdominal wall hernia (*n* = 2). One patient developed nonocclusive mesenteric ischemia and died 35 days after surgery (Clavien–Dindo grade 5). Figure 2a, b show the cumulative proportions of continence recovery in the RARP group (*n* = 500); 458 (91.6%) and 344 (68.8%) patients achieved ≤ 1 pad/day and pad-free, respectively, for urinary continence. In contrast, Fig. 2c, d illustrate the cumulative proportions of grade ≥ 2 GU and GI complications in the VMAT group (*n* = 360); 72 (20.0%) and 16 (4.4%) patients experienced grade ≥ 2 GU and GI complications, respectively. Furthermore, 8 (2.2%) patients developed grade ≥ 2 rectal bleeding (Supplementary Fig. 3). Regarding grade ≥ 3 complications (ten cases in total), one patient had diverticular bleeding (grade 3 GI), whereas five had vesical bleeding and four had urinary retention (all grade 3 GU).

## Discussion

In the present study, we reported 6‑year outcomes of RARP vs. VMAT (a rotational IMRT) for localized PC as the leading respective techniques for prostatectomy and radiotherapy. We compared oncological outcomes, including mortality (OS, CSS, rRFS, and bRFS), between RARP and VMAT using PSM after a follow-up duration of > 6 years (median: 79 months). We also reported complication outcomes of RARP and VMAT after the extended follow-up. RARP and VMAT showed equivalent results for OS, CSS, and rRFS, after PSM. Both methods achieved excellent 6‑year outcomes for OS (> 96%), CSS (> 98%), and rRFS (> 91%). We also presented bRFS results, which, however, should be cautiously interpreted because of different definitions of biochemical recurrence between RARP and VMAT. Regarding complication outcomes, patients who underwent RARP had minimal (2.6%) severe perioperative complications and achieved excellent continence recovery results (91.6 and 68.8% achieved ≤ 1 pad/day and pad-free, respectively). In comparison, patients who underwent VMAT had an acceptable rate (20.0%) of grade ≥ 2 GU complications and very low rates (4.4 and 2.2%) of grade ≥ 2 GI complications and rectal bleeding. These results showed that 6‑year outcomes following RARP and VMAT were comparable and excellent regarding both cancer control and adverse events.

Many studies have compared oncological outcomes of radical prostatectomy and external beam radiotherapy for localized PC [3–18]. For example, outcomes of the ProtecT trial—a large-scale randomized controlled trial—have recently been updated, which showed comparable mortality outcomes for prostatectomy (*n* = 553) and radiotherapy (*n* = 545) as well as active monitoring (*n* = 545) at a median follow-up of 15 years [18]. Most of the other studies were either population-based or retrospective in design [4–17]. Some studies showed equality of the two treatment modalities [4, 6, 7, 11–13], and others suggested a possible survival benefit of prostatectomy over radiotherapy on the basis of long-term observations [5, 8–10, 14–17]. However, given that both treatment modalities have advanced and been improved, comparisons of prostatectomy and radiotherapy should be updated often on the basis of the latest techniques. RARP and IMRT are the leading respective techniques for prostatectomy and radiotherapy, both of which are currently used worldwide. However, almost no study has directly compared outcomes between the techniques. Some studies have compared the outcomes of RARP vs. radiotherapy (not confined to IMRT) [14], and others have compared those of prostatectomy (not confined to RARP) vs. IMRT [6, 7, 13, 15, 16]. Only one study directly compared the outcomes of RARP vs. IMRT. We previously reported the comparative outcomes of RARP versus VMAT in 860 patients with cT1-4N0M0 PC [12]. The study was the first to compare the outcomes of RARP and IMRT. However, the follow-up duration (median: 37 months) was too short to compare mortality outcomes. Therefore, in the present study, we extended the follow-up duration by five years [12] and compared the outcomes of RARP and VMAT, including mortality, using PSM. To our knowledge, our series are currently the only direct comparisons of RARP vs. IMRT.

In addition to oncological outcomes, it is vital to compare complications following prostatectomy and radiotherapy [3]. The ProtecT trial reported patient-reported complication outcomes following prostatectomy and radiotherapy as well as active monitoring at a median follow-up of 10 years [28]. In the trial, prostatectomy had the greatest negative effect on sexual function and urinary continence among the three groups in the study, whereas urinary voiding, nocturia, and bowel function were worse in the radiotherapy group at 6 months compared with the other groups [28]. The present study assessed and elucidated these common complications of prostatectomy and radiotherapy in patients who underwent RARP and VMAT. These treatment-related complications should be considered in the choice of the two modalities as well as their oncological outcomes.

The present study was limited by its retrospective design and relatively small number of events for the mortality endpoints (OS and CSS), as is often the case with clinical studies of PC. Although this study used PSM to reduce bias between the two groups, there might exist unknown variables which could only be adjusted for by randomization. Furthermore, due to the significant differences in baseline patient characteristics between the RARP and VMAT groups, the patient number after PSM became relatively small (*n* = 260), which potentially limited the study’s statistical power. Lastly, this study did not evaluate erectile function which should be an important clinical parameter when comparing RARP vs. VMAT. Although hypofractionated radiotherapy is increasingly used for PC [29], randomized controlled trials with long-term follow-up are warranted to confirm our results of RARP vs. VMAT.

## Conclusion

In this study using PSM after a 6-year follow-up, RARP and VMAT showed equivalent and excellent oncological outcomes, as well as acceptable complication profiles.

### Supplementary Information

**Supplementary Fig. 1. **Kaplan–Meier curves of the RARP vs. VMAT patients for (A) OS, (B) CSS, (C) rRFS, and (D) bRFS in the original cohort before matching (*n* = 860). bRFS, biochemical recurrence-free survival; CSS, cancer-specific survival; OS, overall survival; RARP, robot-assisted radical prostatectomy; rRFS, radiological recurrence-free survival; VMAT, volumetric modulated arc therapy

**Supplementary Fig. 2.** Kaplan–Meier curves of RARP vs. VMAT with < 2-year ADT vs. VMAT with ≥ 2-year ADT for bRFS (A) in the original cohort before matching (*n* = 860) and (B) in the matched cohort (*n* = 260). ADT, androgen deprivation therapy; bRFS, biochemical recurrence-free survival; RARP, robot-assisted radical prostatectomy; VMAT, volumetric modulated arc therapy

**Supplementary Fig. 3. **Cumulative proportion of patients with grade ≥ 2 rectal bleeding in the VMAT group (*n* = 360). VMAT, volumetric modulated arc therapy

**Supplementary Table 1.** Univariate and multivariate Cox proportional hazard regression analyses of (A) OS, (B) rRFS, and (C) bRFS before PSM (*n* = 860).

**Supplementary Table 2.** Univariate and multivariate Cox proportional hazard regression analyses of (A) OS, (B) rRFS, and (C) bRFS after PSM (*n* = 260).

**Supplementary Table 3.** Clavien–Dindo grade ≥ 3 perioperative complications in the RARP group (*n* = 500).

## Data Availability

The datasets used and analyzed during the current study are available from the corresponding author upon reasonable request.

## Abbreviations

ADT: Androgen deprivation therapy
bRFS: Biochemical recurrence-free survival
CCI: Charlson comorbidity index
CSS: Cancer-specific survival
GI: Gastrointestinal
GU: Genitourinary
IMRT: Intensity-modulated radiotherapy
IQR: Interquartile range
OS: Overall survival
PC: Prostate cancer
ProtecT: Prostate Testing for Cancer and Treatment
PSA: Prostate-specific antigen
PSM: Propensity score matching
RARP: Robot-assisted radical prostatectomy
rRFS: Radiological recurrence-free survival
VMAT: Volumetric modulated arc therapy

## References

[1] Mottet N, van den Bergh RCN, Briers E et al (2021) EAU-EANM-ESTRO-ESUR-SIOG Guidelines on Prostate Cancer—2020 Update. Part 1: Screening, Diagnosis, and Local Treatment with Curative Intent. Eur Urol 79:243–262 33172724 10.1016/j.eururo.2020.09.042

[2] Kakehi Y, Sugimoto M, Taoka R; committee for establishment of the evidenced-based clinical practice guideline for prostate cancer of the Japanese Urological Association (2017) Evidenced-based clinical practice guideline for prostate cancer (summary: Japanese Urological Association, 2016 edition). Int J Urol 24:648–666. 10.1111/iju.13380 28667698

[3] Taguchi S, Shiraishi K, Fukuhara H (2020) Updated evidence on oncological outcomes of surgery versus external beam radiotherapy for localized prostate cancer. Jpn J Clin Oncol 50:963–969 32580211 10.1093/jjco/hyaa105

[4] D’Amico AV, Whittington R, Malkowicz SB et al (1998) Biochemical outcome after radical prostatectomy, external beam radiation therapy, or interstitial radiation therapy for clinically localized prostate cancer. JAMA 280:969–974 9749478 10.1001/jama.280.11.969

[5] Merglen A, Schmidlin F, Fioretta G et al (2007) Short- and long-term mortality with localized prostate cancer. Arch Intern Med 167:1944–1950 17923593 10.1001/archinte.167.18.1944

[6] Aizer AA, Yu JB, Colberg JW et al (2009) Radical prostatectomy vs. intensity-modulated radiation therapy in the management of localized prostate adenocarcinoma. Radiother Oncol 93:185–191 19800702 10.1016/j.radonc.2009.09.001

[7] Merino T, San Francisco IF, Rojas PA et al (2013) Intensity-modulated radiotherapy versus radical prostatectomy in patients with localized prostate cancer: long-term follow-up. BMC Cancer 13:530 24209381 10.1186/1471-2407-13-530 PMC3833713

[8] Hoffman RM, Koyama T, Fan KH et al (2013) Mortality after radical prostatectomy or external beam radiotherapy for localized prostate cancer. J Natl Cancer Inst 105:711–718 23615689 10.1093/jnci/djt059 PMC3653822

[9] Sooriakumaran P, Nyberg T, Akre O et al (2014) Comparative effectiveness of radical prostatectomy and radiotherapy in prostate cancer: observational study of mortality outcomes. BMJ 348:g1502 24574496 10.1136/bmj.g1502 PMC3936107

[10] Taguchi S, Fukuhara H, Shiraishi K et al (2015) Radical Prostatectomy versus External Beam Radiotherapy for cT1-4N0M0 Prostate Cancer: Comparison of Patient Outcomes Including Mortality. PLoS ONE 10:e141123 26506569 10.1371/journal.pone.0141123 PMC4624690

[11] Kishan AU, Cook RR, Ciezki JP et al (2018) Radical prostatectomy, external beam radiotherapy, or external beam radiotherapy with brachytherapy boost and disease progression and mortality in patients with Gleason score 9–10 prostate cancer. JAMA 319:896–905 29509865 10.1001/jama.2018.0587 PMC5885899

[12] Taguchi S, Shiraishi K, Fujimura T et al (2019) Robot-assisted radical prostatectomy versus volumetric modulated arc therapy: Comparison of front-line therapies for localized prostate cancer. Radiother Oncol 140:62–67 31176208 10.1016/j.radonc.2019.05.015

[13] Hayashi N, Osaka K, Muraoka K et al (2020) Outcomes of treatment for localized prostate cancer in a single institution: comparison of radical prostatectomy and radiation therapy by propensity score matching analysis. World J Urol 38:2477–2484 31875247 10.1007/s00345-019-03056-3

[14] Ko YH (2021) The comparison of the survival outcome between robotic-assisted radical prostatectomy and radiation therapy for localized prostate cancer in men over 70 years: Korean Nationwide Observational Study. J Robot Surg 15:585–592 32918235 10.1007/s11701-020-01144-w

[15] Wu SY, Chang SC, Chen CI et al (2021) Oncologic Outcomes of Radical Prostatectomy and High-Dose Intensity-Modulated Radiotherapy with Androgen-Deprivation Therapy for Relatively Young Patients with Unfavorable Intermediate-Risk Prostate Adenocarcinoma. Cancers (Basel) 13:1517 33806181 10.3390/cancers13071517 PMC8036838

[16] Wu SY, Effendi FF, Canales RE et al (2022) The Latest Data Specifically Focused on Long-Term Oncologic Prognostication for Very Old Adults with Acute Vulnerable Localized Prostate Cancer: A Nationwide Cohort Study. J Clin Med 11:3451 35743522 10.3390/jcm11123451 PMC9225393

[17] Chierigo F, Wenzel M, Würnschimmel C et al (2022) Survival after Radical Prostatectomy versus Radiation Therapy in High-Risk and Very High-Risk Prostate Cancer. J Urol 207:375–384 34555930 10.1097/JU.0000000000002250

[18] Hamdy FC, Donovan JL, Lane JA, ProtecT Study Group (2023) Fifteen-Year Outcomes after Monitoring or Radiotherapy for Prostate Cancer. N Engl J Med 388:1547–1558 10.1056/NEJMoa2214122 36912538

[19] Wolff D, Stieler F, Welzel G et al (2009) Volumetric modulated arc therapy (VMAT) vs. serial tomotherapy, step-and-shoot IMRT and 3D-conformal RT for treatment of prostate cancer. Radiother Oncol 93:226–233 19765846 10.1016/j.radonc.2009.08.011

[20] Charlson ME, Pompei P, Ales KL et al (1987) A new method of classifying prognostic comorbidity in longitudinal studies: development and validation. J Chronic Dis 40:373–383 3558716 10.1016/0021-9681(87)90171-8

[21] Shrivastava A, Baliga M, Menon M (2007) The Vattikuti Institute prostatectomy. BJU Int 99:1173–1189 17437452 10.1111/j.1464-410X.2007.06878.x

[22] Fujimura T, Menon M, Fukuhara H et al (2016) Validation of an educational program balancing surgeon training and surgical quality control during robot-assisted radical prostatectomy. Int J Urol 23:160–166 26502293 10.1111/iju.12993

[23] Naito S, Kuroiwa K, Kinukawa N, et al; Clinicopathological Research Group for Localized Prostate Cancer Investigators (2008) Validation of Partin tables and development of a preoperative nomogram for Japanese patients with clinically localized prostate cancer using 2005 International Society of Urological Pathology consensus on Gleason grading: data from the clinicopathological research group for localized prostate cancer. J Urol 180:904–909. 10.1016/j.juro.2008.05.047 18635221

[24] Roach M 3rd, Hanks G, Thames H Jr et al (2006) Defining biochemical failure following radiotherapy with or without hormonal therapy in men with clinically localized prostate cancer: recommendations of the RTOG-ASTRO phoenix consensus conference. Int J Radiat Oncol Biol Phys 65:965–974 16798415 10.1016/j.ijrobp.2006.04.029

[25] Clavien PA, Sanabria JR, Strasberg SM (1992) Proposed classification of complications of surgery with examples of utility in cholecystectomy. Surgery 111:518–526 1598671

[26] Dindo D, Demartines N, Clavien PA (2004) Classification of surgical complications: a new proposal with evaluation in a cohort of 6336 patients and results of a survey. Ann Surg 240:205–213 15273542 10.1097/01.sla.0000133083.54934.ae PMC1360123

[27] Yamada Y, Teshima T, Fujimura T et al (2020) Comparison of perioperative outcomes in elderly (age ≧ 75 years) vs. younger men undergoing robot-assisted radical prostatectomy. PLoS ONE 15:e234113 32497131 10.1371/journal.pone.0234113 PMC7272059

[28] Donovan JL, Hamdy FC, Lane JA, ProtecT Study Group (2016) Patient-Reported Outcomes after Monitoring or Radiotherapy for Prostate Cancer. N Engl J Med 375:1425–1437 10.1056/NEJMoa1606221 PMC5134995 27626365

[29] Tree AC, Ostler P, van der Voet H, PACE Trial Investigators (2022) Intensity-modulated radiotherapy versus stereotactic body radiotherapy for prostate cancer (PACE-B): 2‑year toxicity results from an open-label phase 3, non-inferiority trial. Lancet Oncol 23:1308–1320 10.1016/S1470-2045(22)00517-4 36113498

