# Supplementary material for: Six-year outcomes of robot-assisted radical prostatectomy versus volumetric modulated arc therapy for localized prostate cancer: A propensity score-matched analysis
Source: Strahlenther Onkol. 2024 Jan 5;200(8):676–83. doi: 10.1007/s00066-023-02192-5 (PMC11272719; doi:10.1007/s00066-023-02192-5)
Supplement: Supplementary file 1 — Supplementary Fig. 1. Kaplan–Meier curves of the RARP vs. VMAT patients for (A) OS, (B) CSS, (C) rRFS, and (D) bRFS in the original cohort before matching (n = 860). bRFS, biochemical recurrence-free survival; CSS, cancer-specific survival; OS, overall survival; RARP, robot-assisted radical prostatectomy; rRFS, radiological recurrence-free survival; VMAT, volumetric modulated arc therapy [file 66_2023_2192_MOESM1_ESM.pdf]

(A) Original cohort (before matching; *n* = 860)

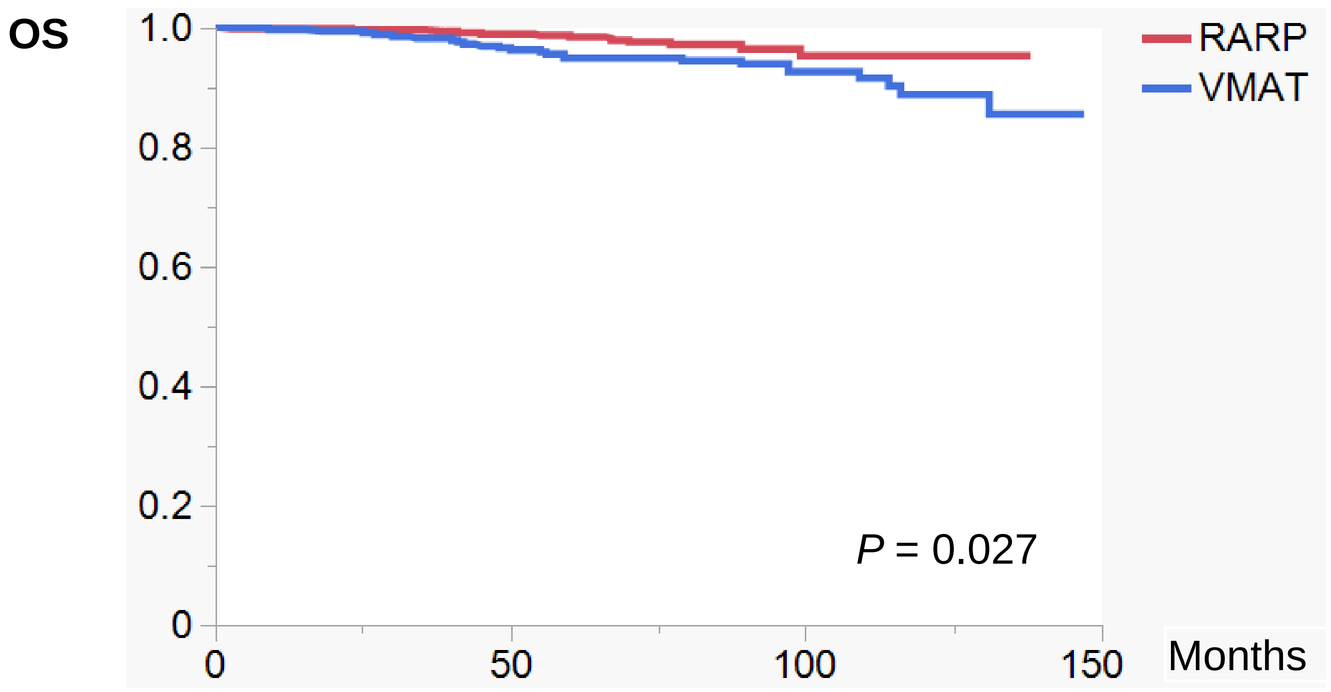

Number at risk:

RARP	500	439	77	0
VMAT	360	296	114	0

(B) Original cohort (before matching; *n* = 860)

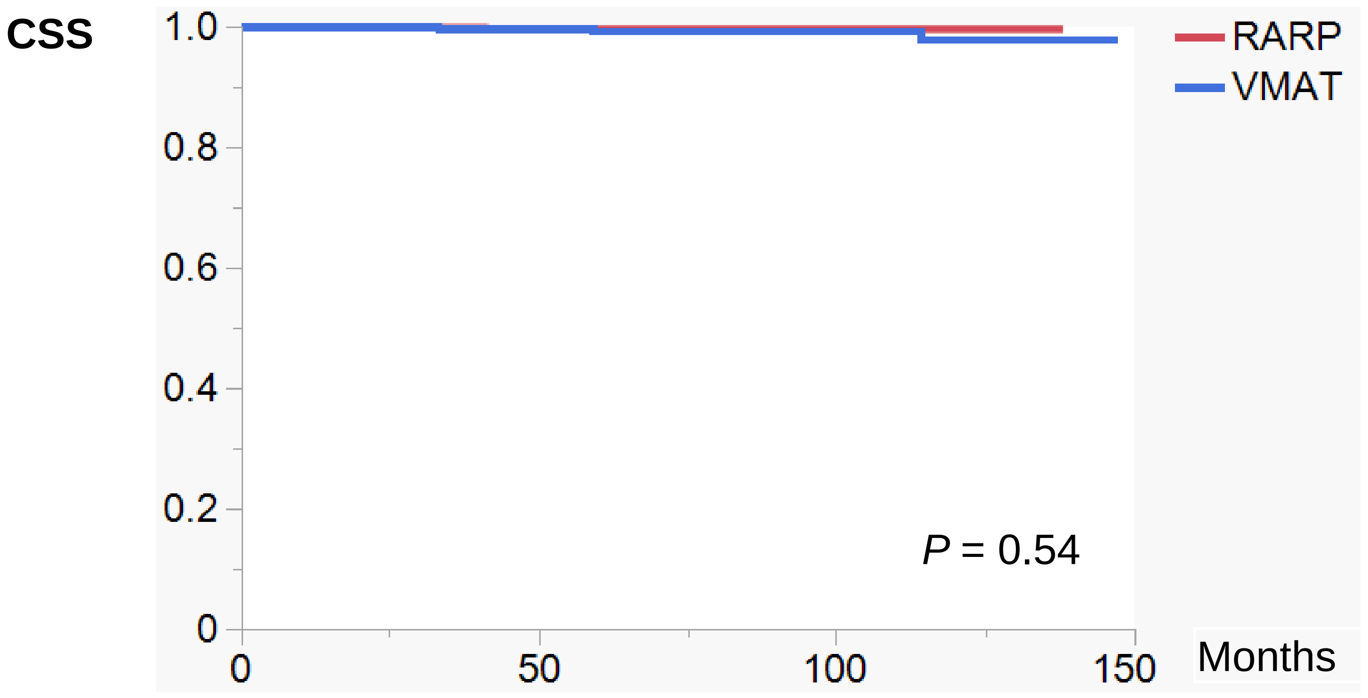

Number at risk:

RARP	500	439	77	0
VMAT	360	296	114	0

(C) Original cohort (before matching; *n* = 860)

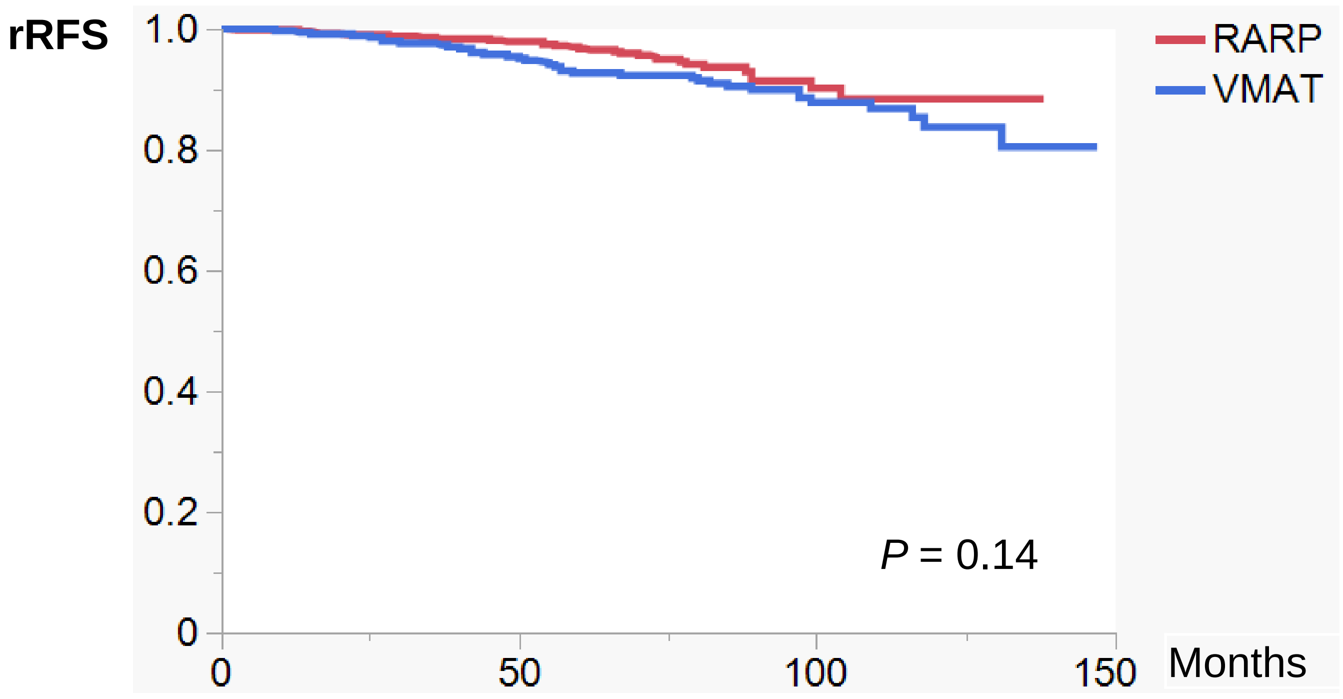

Number at risk:

RARP	500	434	71	0
VMAT	360	291	107	0

(D) Original cohort (before matching; *n* = 860)

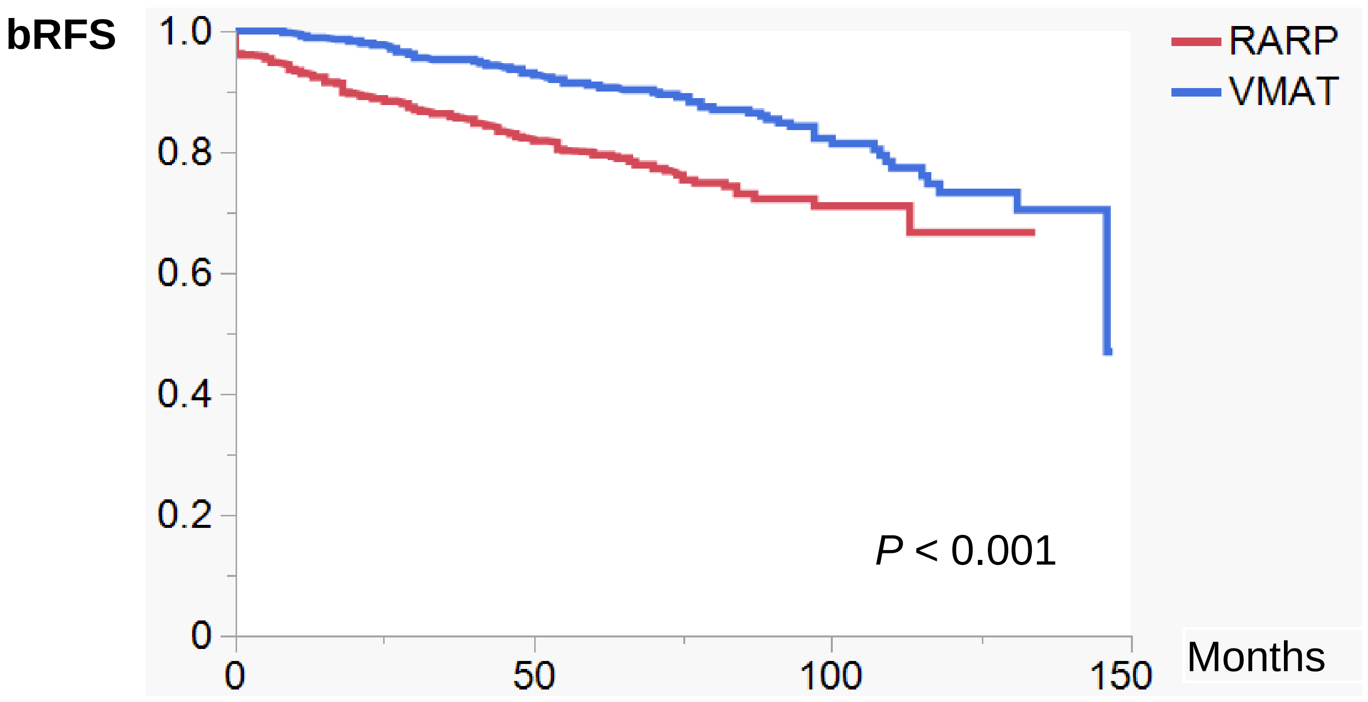

Number at risk:

RARP	500	360	46	0
VMAT	360	284	99	0
