# Supplementary material for: Six-year outcomes of robot-assisted radical prostatectomy versus volumetric modulated arc therapy for localized prostate cancer: A propensity score-matched analysis
Source: Strahlenther Onkol. 2024 Jan 5;200(8):676–83. doi: 10.1007/s00066-023-02192-5 (PMC11272719; doi:10.1007/s00066-023-02192-5)
Supplement: Supplementary file 2 — Supplementary Fig. 2. Kaplan–Meier curves of RARP vs. VMAT with < 2-year ADT vs. VMAT with ≥ 2-year ADT for bRFS (A) in the original cohort before matching (n = 860) and (B) in the matched cohort (n = 260). ADT, androgen deprivation therapy; bRFS, biochemical recurrence-free survival; RARP, robot-assisted radical prostatectomy; VMAT, volumetric modulated arc therapy [file 66_2023_2192_MOESM2_ESM.pdf]

(A) Original cohort (before matching; n = 860)

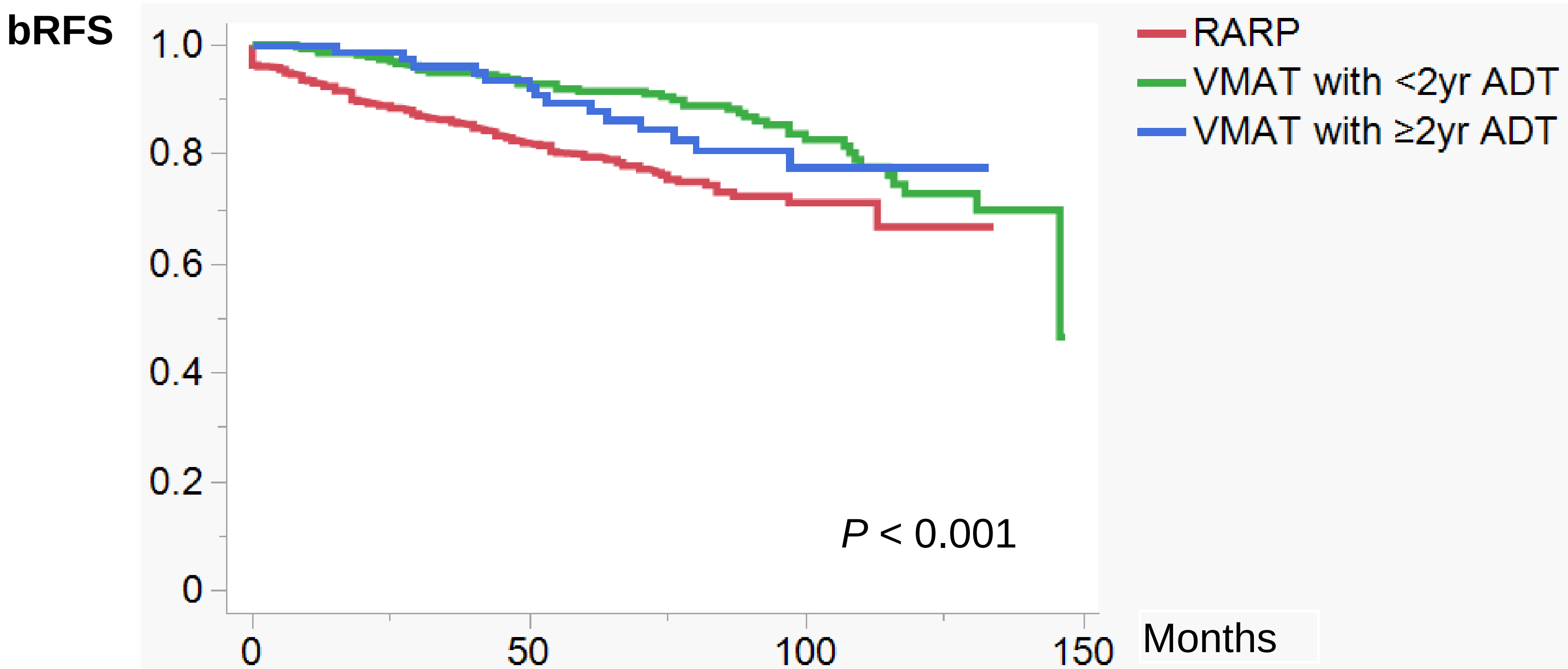

Number at risk:

RARP	500	360	46	0
VMAT with <2yr ADT	272	214	82	0
VMAT with ≥2yr ADT	88	70	17	0

P < 0.001 (RARP vs. VMAT with <2yr ADT)
P = 0.55 (VMAT with <2yr ADT vs. VMAT with ≥2yr ADT)
P = 0.071 (RARP vs. VMAT with ≥2yr ADT)

(B) Matched cohort (n = 260)

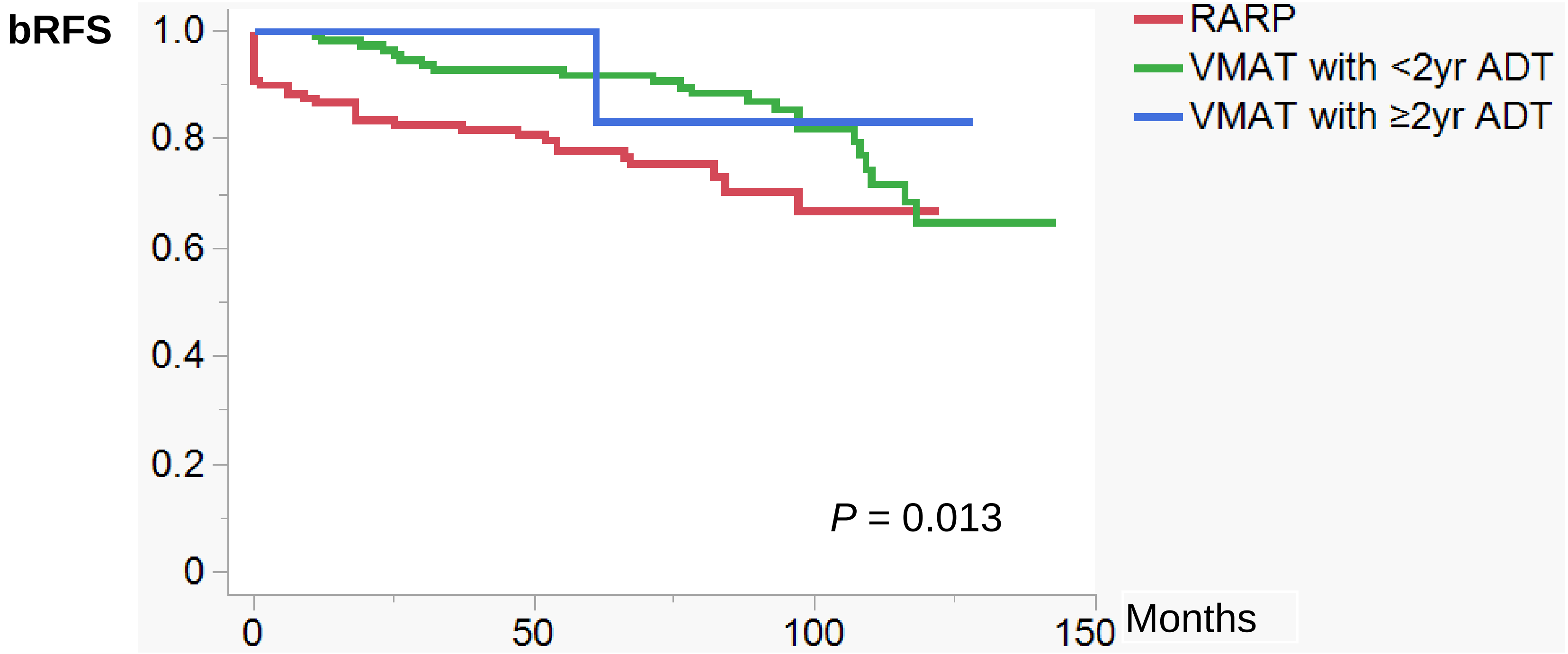

Number at risk:

RARP	130	85	11	0
VMAT with <2yr ADT	122	95	39	0
VMAT with ≥2yr ADT	8	7	2	0

P = 0.004 (RARP vs. VMAT with <2yr ADT)
P = 0.75 (VMAT with <2yr ADT vs. VMAT with ≥2yr ADT)
P = 0.37 (RARP vs. VMAT with ≥2yr ADT)
