# Supplementary figures and images for: Six-year outcomes of robot-assisted radical prostatectomy versus volumetric modulated arc therapy for localized prostate cancer: A propensity score-matched analysis
Source: Strahlenther Onkol. 2024 Jan 5;200(8):676–83. doi: 10.1007/s00066-023-02192-5 (PMC11272719; doi:10.1007/s00066-023-02192-5)

## Grade $\geq 2$ rectal bleeding

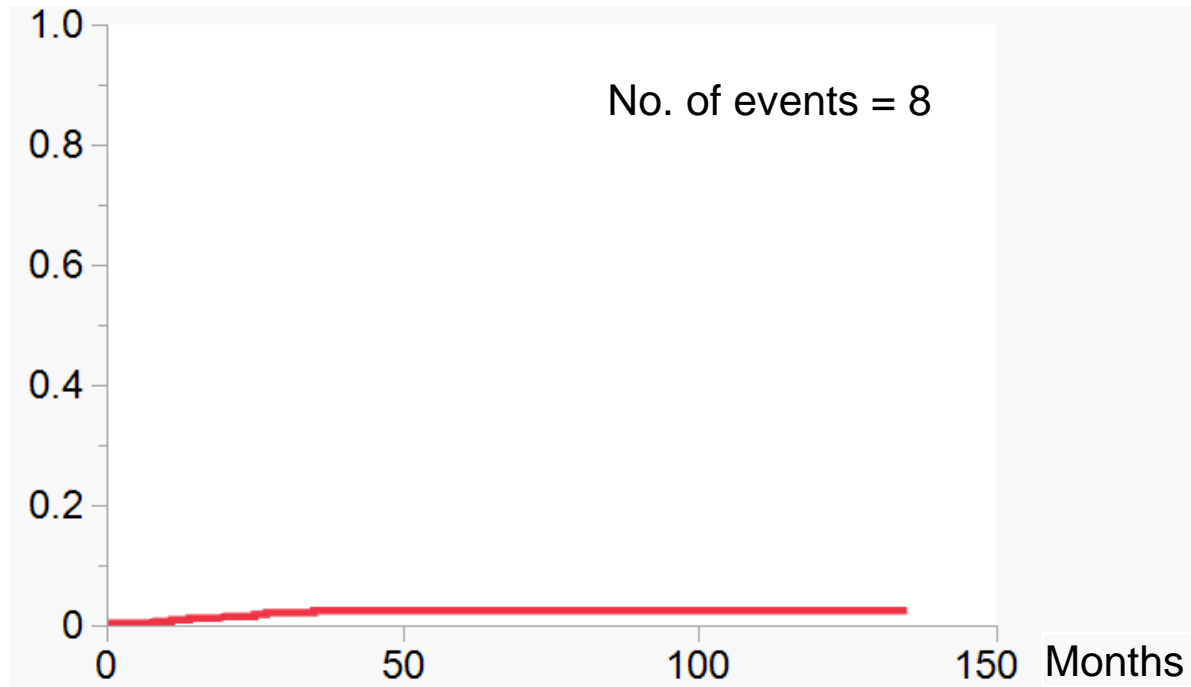

Number at risk:

| VMAT           | 360 | 242 | 12 | 0 |
|----------------|-----|-----|----|---|
| Number at risk | 360 | 242 | 12 | 0 |

Supplement: Supplementary file 3 — Supplementary Fig. 3. Cumulative proportion of patients with grade ≥ 2 rectal bleeding in the VMAT group (n = 360). VMAT, volumetric modulated arc therapy [file 66_2023_2192_MOESM3_ESM.pdf]
