# Supplementary material for: Six-year outcomes of robot-assisted radical prostatectomy versus volumetric modulated arc therapy for localized prostate cancer: A propensity score-matched analysis
Source: Strahlenther Onkol. 2024 Jan 5;200(8):676–83. doi: 10.1007/s00066-023-02192-5 (PMC11272719; doi:10.1007/s00066-023-02192-5)
Supplement: Supplementary file 4 — Supplementary Table 1. Univariate and multivariate Cox proportional hazard regression analyses of (A) OS, (B) rRFS, and (C) bRFS before PSM (n = 860). [file 66_2023_2192_MOESM4_ESM.docx]

**Supplementary Table 1.** Univariate and multivariate Cox proportional hazard regression analyses of (A) OS, (B) rRFS, and (C) bRFS before PSM (*n* = 860).

(A) Univariate and multivariate Cox proportional hazard regression analyses of OS before PSM (*n* = 860).

| Parameter | Univariate | | Multivariate | |
| --- | --- | --- | --- | --- |
|  | HR (95% CI) | *P* | HR (95% CI) | *P* |
| Treatment modality |  |  |  |  |
| VMAT (vs. RARP) | 2.14 (1.07 to 4.25) | 0.031^*^ | 1.26 (0.48 to 3.31) | 0.64 |
| D’Amico risk classification |  | 0.004^*^ |  | 0.041^*^ |
| Intermediate (vs. Low) | 0.85 (0.23 to 3.10) | 0.81 | 0.82 (0.22 to 3.00) | 0.76 |
| High (vs. Low) | 2.76 (0.83 to 9.16) | 0.098 | 2.39 (0.68 to 8.45) | 0.18 |
| High (vs. Intermediate) | 3.24 (1.55 to 6.79) | 0.002^*^ | 2.92 (1.23 to 6.90) | 0.015^*^ |
| Age-adjusted CCI |  |  |  |  |
| Continuous | 1.48 (1.23 to 1.74) per score | < 0.001^*^ | 1.39 (1.13 to 1.67) per score | 0.002^*^ |
| Concomitant ADT |  |  |  |  |
| Yes (vs. No) | 2.26 (1.17 to 4.33) | 0.015^*^ | 0.82 (0.30 to 2.20) | 0.69 |

ADT, androgen deprivation therapy; CCI, Charlson comorbidity index; CI, confidence interval; HR, hazard ratio; OS, overall survival; PSM, propensity score matching; RARP, robot-assisted radical prostatectomy; VMAT, volumetric modulated arc therapy

^*^ Statistically significant

(B) Univariate and multivariate Cox proportional hazard regression analyses of rRFS before PSM (*n* = 860).

| Parameter | Univariate | | Multivariate | |
| --- | --- | --- | --- | --- |
|  | HR (95% CI) | *P* | HR (95% CI) | *P* |
| Treatment modality |  |  |  |  |
| VMAT (vs. RARP) | 1.45 (0.88 to 2.39) | 0.14 | 0.62 (0.28 to 1.34) | 0.22 |
| D’Amico risk classification |  | < 0.001^*^ |  | < 0.001^*^ |
| Intermediate (vs. Low) | 1.34 (0.39 to 4.60) | 0.64 | 1.33 (0.39 to 4.58) | 0.65 |
| High (vs. Low) | 5.49 (1.71 to 17.65) | 0.004^*^ | 4.47 (1.35 to 14.79) | 0.014^*^ |
| High (vs. Intermediate) | 4.10 (2.32 to 7.24) | < 0.001^*^ | 3.35 (1.77 to 6.36) | < 0.001^*^ |
| Age-adjusted CCI |  |  |  |  |
| Continuous | 1.35 (1.16 to 1.56) per score | < 0.001^*^ | 1.29 (1.09 to 1.51) per score | 0.004^*^ |
| Concomitant ADT |  |  |  |  |
| Yes (vs. No) | 2.47 (1.51 to 4.04) | < 0.001^*^ | 1.46 (0.66 to 3.23) | 0.35 |

ADT, androgen-deprivation therapy; CCI, Charlson comorbidity index; CI, confidence interval; HR, hazard ratio; PSM, propensity score matching; RARP, robot-assisted radical prostatectomy; rRFS, radiological recurrence-free survival; VMAT, volumetric modulated arc therapy

^*^ Statistically significant

(C) Univariate and multivariate Cox proportional hazard regression analyses of bRFS before PSM (*n* = 860).

| Parameter | Univariate | | Multivariate | |
| --- | --- | --- | --- | --- |
|  | HR (95% CI) | *P* | HR (95% CI) | *P* |
| Treatment modality |  |  |  |  |
| VMAT (vs. RARP) | 0.50 (0.36 to 0.70) | < 0.001^*^ | 0.23(0.13 to 0.40) | < 0.001^*^ |
| D’Amico risk classification |  | < 0.001^*^ |  | < 0.001^*^ |
| Intermediate (vs. Low) | 2.52 (1.21 to 5.22) | 0.013^*^ | 2.55 (1.23 to 5.28) | 0.012^*^ |
| High (vs. Low) | 4.36 (2.11 to 8.98) | < 0.001^*^ | 4.60 (2.20 to 9.61) | < 0.001^*^ |
| High (vs. Intermediate) | 1.73 (1.27 to 2.35) | < 0.001^*^ | 1.81 (1.29 to 2.53) | < 0.001^*^ |
| Age-adjusted CCI |  |  |  |  |
| Continuous | 0.97 (0.86 to 1.09) per score | 0.62 | 1.08 (0.95 to 1.22) per score | 0.27 |
| Concomitant ADT |  |  |  |  |
| Yes (vs. No) | 1.02 (0.74 to 1.42) | 0.88 | 2.03 (1.17 to 3.52) | 0.011^*^ |

ADT, androgen deprivation therapy; bRFS, biochemical recurrence-free survival; CCI, Charlson comorbidity index; CI, confidence interval; HR, hazard ratio; PSA, prostate-specific antigen; PSM, propensity score matching; RARP, robot-assisted radical prostatectomy; VMAT, volumetric modulated arc therapy

^*^ Statistically significant
