# Supplementary material for: Six-year outcomes of robot-assisted radical prostatectomy versus volumetric modulated arc therapy for localized prostate cancer: A propensity score-matched analysis
Source: Strahlenther Onkol. 2024 Jan 5;200(8):676–83. doi: 10.1007/s00066-023-02192-5 (PMC11272719; doi:10.1007/s00066-023-02192-5)
Supplement: Supplementary file 5 — Supplementary Table 2. Univariate and multivariate Cox proportional hazard regression analyses of (A) OS, (B) rRFS, and (C) bRFS after PSM (n = 260). [file 66_2023_2192_MOESM5_ESM.docx]

**Supplementary Table 2.** Univariate and multivariate Cox proportional hazard regression analyses of (A) OS, (B) rRFS, and (C) bRFS after PSM (*n* = 260).

(A) Univariate and multivariate Cox proportional hazard regression analyses of OS after PSM (*n* = 260).

| Parameter | Univariate | | Multivariate | |
| --- | --- | --- | --- | --- |
|  | HR (95% CI) | *P* | HR (95% CI) | *P* |
| Treatment modality |  |  |  |  |
| VMAT (vs. RARP) | 1.33 (0.38 to 4.71) | 0.65 | 1.41 (0.39 to 5.02) | 0.60 |
| D’Amico risk classification |  | 0.24 |  | 0.22 |
| Intermediate (vs. Low) | 0.56 (0.10 to 3.09) | 0.51 | 0.55 (0.099 to 3.03) | 0.49 |
| High (vs. Low) | 1.75 (0.34 to 9.05) | 0.50 | 2.24 (0.34 to 14.80) | 0.40 |
| High (vs. Intermediate) | 3.11 (0.83 to 11.68) | 0.092 | 4.10 (0.83 to 20.26) | 0.084 |
| Age-adjusted CCI |  |  |  |  |
| Continuous | 1.07 (0.55 to 1.97) per score | 0.83 | 1.17 (0.56 to 2.33) per score | 0.67 |
| Concomitant ADT |  |  |  |  |
| Yes (vs. No) | 1.52 (0.40 to 5.74) | 0.54 | 0.71 (0.12 to 4.08) | 0.70 |

ADT, androgen deprivation therapy; CCI, Charlson comorbidity index; CI, confidence interval; HR, hazard ratio; OS, overall survival; PSM, propensity score matching; RARP, robot-assisted radical prostatectomy; VMAT, volumetric modulated arc therapy

(B) Univariate and multivariate Cox proportional hazard regression analyses of rRFS after PSM (*n* = 260).

| Parameter | Univariate | | Multivariate | |
| --- | --- | --- | --- | --- |
|  | HR (95% CI) | *P* | HR (95% CI) | *P* |
| Treatment modality |  |  |  |  |
| VMAT (vs. RARP) | 0.69 (0.28 to 1.66) | 0.40 | 0.67 (0.28 to 1.63) | 0.38 |
| D’Amico risk classification |  | 0.007^*^ |  | 0.41 |
| Intermediate (vs. Low) | 1.09 (0.23 to 5.14) | 0.91 | 1.07 (0.23 to 5.05) | 0.94 |
| High (vs. Low) | 4.31 (0.95 to 19.4) | 0.058 | 2.42 (0.41 to 14.32) | 0.33 |
| High (vs. Intermediate) | 3.95 (1.58 to 9.84) | 0.003^*^ | 2.27 (0.65 to 7.92) | 0.20 |
| Age-adjusted CCI |  |  |  |  |
| Continuous | 0.80 (0.49 to 1.28) per score | 0.36 | 0.98 (0.58 to 1.60) per score | 0.94 |
| Concomitant ADT |  |  |  |  |
| Yes (vs. No) | 4.10 (1.74 to 9.65) | 0.001^*^ | 2.26 (0.63 to 8.18) | 0.21 |

ADT, androgen deprivation therapy; CCI, Charlson comorbidity index; CI, confidence interval; HR, hazard ratio; PSM, propensity score matching; RARP, robot-assisted radical prostatectomy; rRFS, radiological recurrence-free survival; VMAT, volumetric modulated arc therapy

^*^ Statistically significant

(C) Univariate and multivariate Cox proportional hazard regression analyses of bRFS after PSM (*n* = 260).

| Parameter | Univariate | | Multivariate | |
| --- | --- | --- | --- | --- |
|  | HR (95% CI) | *P* | HR (95% CI) | *P* |
| Treatment modality |  |  |  |  |
| VMAT (vs. RARP) | 0.44 (0.25 to 0.77) | 0.004^*^ | 0.34 (0.19 to 0.61) | < 0.001^*^ |
| D’Amico risk classification |  | < 0.001^*^ |  | 0.34 |
| Intermediate (vs. Low) | 1.54 (0.59 to 4.00) | 0.38 | 1.45 (0.56 to 3.80) | 0.44 |
| High (vs. Low) | 4.24 (1.61 to 11.16) | 0.004^*^ | 2.21 (0.73 to 6.70) | 0.16 |
| High (vs. Intermediate) | 2.75 (1.57 to 4.82) | < 0.001^*^ | 1.52 (0.73 to 3.16) | 0.27 |
| Age-adjusted CCI |  |  |  |  |
| Continuous | 0.91 (0.68 to 1.21) per score | 0.52 | 1.13 (0.84 to 1.52) per score | 0.44 |
| Concomitant ADT |  |  |  |  |
| Yes (vs. No) | 3.75 (2.18 to 6.44) | < 0.001^*^ | 3.64 (1.67 to 7.92) | 0.001^*^ |

ADT, androgen deprivation therapy; bRFS, biochemical recurrence-free survival; CCI, Charlson comorbidity index; CI, confidence interval; HR, hazard ratio; PSA, prostate-specific antigen; PSM, propensity score matching; RARP, robot-assisted radical prostatectomy; VMAT, volumetric modulated arc therapy

^*^ Statistically significant
