# Supplementary material for: Six-year outcomes of robot-assisted radical prostatectomy versus volumetric modulated arc therapy for localized prostate cancer: A propensity score-matched analysis
Source: Strahlenther Onkol. 2024 Jan 5;200(8):676–83. doi: 10.1007/s00066-023-02192-5 (PMC11272719; doi:10.1007/s00066-023-02192-5)
Supplement: Supplementary file 6 — Supplementary Table 3. Clavien–Dindo grade ≥ 3 perioperative complications in the RARP group (n = 500). [file 66_2023_2192_MOESM6_ESM.docx]

**Supplementary Table 3.** Clavien-Dindo grade ≥ 3 perioperative complications in the RARP group (*n* = 500).

| Postoperative hemorrhage | 3 |
| --- | --- |
| Rectal injury | 2 |
| Small bowel injury | 2 |
| Abdominal wall hernia | 2 |
| Bladder injury | 1 |
| Urinary leakage | 1 |
| Ileus | 1 |
| Non-occlusive mesenteric ischemia^†^ | 1 |
| Total | 13 |

RARP, robot-assisted radical prostatectomy

^†^ A patient who developed non-occlusive mesenteric ischemia died 35 days after surgery (Clavien-Dindo grade 5).
